# Comprehensive Risk Evaluation of Perfluoroalkyl Substance Pollution in Urban Riverine Systems: Ecotoxicological and Human Health Perspectives

**DOI:** 10.3390/toxics13060435

**Published:** 2025-05-26

**Authors:** Ferlian Vida Satriaji, Cat Tuong Le Tong, Nelly Marlina, Yan Lin, Nguyen Duy Dat, Ha Manh Bui, Yoshifumi Horie, Jheng-Jie Jiang

**Affiliations:** 1 Advanced Environmental Ultra Research Laboratory (ADVENTURE), Department of Environmental Engineering, Chung Yuan Christian University, Taoyuan 320314, Taiwan; 2 Department of Civil Engineering, Chung Yuan Christian University, Taoyuan 320314, Taiwan; 3 School of Environmental Science and Engineering, Xiamen University of Technology, Xiamen 361021, China; 4 Faculty of Chemical and Food Technology, Ho Chi Minh City University of Technology and Education, Ho Chi Minh City 700000, Vietnam; 5 Faculty of Engineering and Technology, Saigon University, Ho Chi Minh City 700000, Vietnam; 6 Research Center for Inland Seas (KURCIS), Kobe University, Fukaeminami-Machi, Higashinada-Ku, Kobe 658-0022, Japan; horie@people.kobe-u.ac.jp; 7 Center for Environmental Risk Management (CERM), Chung Yuan Christian University, Taoyuan 320314, Taiwan; 8 Research Center for Carbon Neutrality and Net Zero Emissions, Chung Yuan Christian University, Taoyuan 320314, Taiwan

**Keywords:** PFASs, occurrence, seasonal variation, risk assessment, source identification

## Abstract

This study investigated the spatiotemporal distribution of perfluoroalkyl substances (PFASs) in the Daku River, Taoyuan, with a particular focus on source apportionment and associated ecological and human health risks. The total PFAS concentrations ranged from below the detection limits to 185 ng/L, with perfluorooctanoic acid (PFOA) emerging as the predominant compound, followed by perfluorobutanesulfonic acid (PFBS). Elevated PFAS levels were observed downstream of the confluence between the Daku River and Litouzhou ditch, suggesting contributions from industrial activities. Principal component analysis (PCA) and positive matrix factorization (PMF) were employed to identify important components and factors that explain different compounds. Factor 1 (dominated by PFUnA) was attributed to sources such as food packaging and textiles. Factor 2 (PFBS, PFHxS, PFOS) originated from agricultural inputs and wastewater discharges linked to the semiconductor and photonics industries. Factor 3 (PFOA, PFNA, PFDA) was primarily associated with fluoropolymer manufacturing, electronics, chemical engineering, machinery, and coating production. Ecological risk assessments showed no significant threats (RQ < 0.1) for PFBS, PFPA, PFNA, PFOS, and PFDA. Human health risk evaluations based on the Health Risk Index (HRI < 1), likewise, indicated negligible risk from crop and vegetable consumption in the Daku River area. These findings underscore the importance of continued monitoring and targeted pollution management strategies to safeguard environmental quality and public health.

## 1. Introduction

Since the 1950s, a class of human-made organic compounds known as perfluoroalkyl substances (PFASs) has been extensively utilized in various industrial applications and consumer goods. These fluorinated chemicals are characterized by their synthetic nature and widespread use. Their exceptional hydrophobic and oleophobic properties, combined with high chemical and thermal stability, and low surface free energy, have led to their extensive use in applications such as coatings, firefighting foams, and textiles [1,2,3]. Structurally, PFASs can be straight- or branched-chain hydrocarbons, wherein most or all hydrogen atoms on carbon bonds are replaced by fluorine atoms, forming stable C–F covalent bonds [4,5,6]. Their resulting high persistence enables PFASs to accumulate in biota and remain in the environment for extended periods.

The health risks associated with PFASs have been increasingly recognized, particularly for legacy compounds such as perfluorooctanoic acid (PFOA) and perfluorooctane sulfonic acid (PFOS). Excessive exposure to these substances has been linked to thyroid disorders, kidney and testicular cancer [7], immune system dysfunction, reduced fertility, and developmental complications in fetuses [8]. Given their persistence, bioaccumulation potential, and capacity for long-range transport, PFASs are classified as persistent organic pollutants (POPs) and subject to restriction under the Stockholm Convention [9]. In addition, manufacturing trends indicate that some PFAS production facilities have been relocated to developing countries or regions with less stringent regulations [10].

Recent research has emphasized the detection and behavior of PFAS in aquatic environments due to their documented negative impacts on both human health and ecological systems. Legacy PFASs, notably PFOA and PFOS, have been frequently detected in riverine systems worldwide, including the Yangtze River (China) [11], Tokyo Bay (Japan) [12], the Langat River (Malaysia) [13], and the Upper Mississippi River (USA) [14]. Despite the breadth of studies on PFAS occurrences, information remains limited regarding PFAS contamination and potential ecological risks in canals and rivers used for agricultural irrigation—an issue of particular concern in Taiwan, where canal and river pollution may have a substantial impact on agricultural production.

The Taoyuan Daku River watershed, situated in the Zhongli, Guanyin, and Xinya districts of Taoyuan City, Taiwan, is primarily utilized in conjunction with the Taoyuan Canal for agricultural irrigation. In this watershed, farmland accounts for approximately 78% of the total area. The Guanyin District, in particular, is a significant rice-producing region in Taoyuan. However, as modern consumption patterns shift and imported agricultural products exert market pressure, some traditional rice fields have been repurposed for organic agriculture or leisure farming. Hence, understanding the levels of PFASs in the Daku River is pivotal, as contaminants may accumulate in crops and potentially pose health risks to consumers. Further compounding these concerns, the downstream region of the Daku River neighbors the Guanyin Industrial Park, hosting numerous light, heavy, and high-tech industries that may release PFASs into the environment.

Accordingly, the objectives of this study were to (1) characterize the spatial distribution of PFASs at upstream, midstream, and downstream sections of the Daku River; (2) elucidate temporal changes in PFAS concentrations through multi-season sampling over an extended period; and (3) evaluate the potential environmental and human exposure risks associated with PFASs in the river’s surface water.

## 2. Materials and Methods

### 2.1. Chemicals and Standards

Analytical-grade chemicals and standards were employed throughout the study. Absolute Standards (Hamden, CT, USA) supplied the target PFAS analytes, which included perfluorobutyl sulfonate (PFBS), perfluorohexyl sulfonate (PFHxS), perfluorooctyl sulfonate (PFOS), perfluoroheptanoic acid (PFHpA), perfluorooctanoic acid (PFOA), perfluorononanoic acid (PFNA), perfluorodecanoic acid (PFDA), perfluoroundecanoic acid (PFUnA), and perfluorododecanoic acid (PFDoA). Cambridge Isotope Laboratories (Andover, MA, USA) provided two isotopically labeled standards: ^13^C_8_-perfluorooctanoic acid (^13^C_8_-PFOA) and ^13^C_9_-perfluorononanoic (^13^C_9_-PFNA). LC-MS-grade methanol was sourced from J.T. Baker (Phillipsburg, NJ, USA), while experimental water was purified using a Milli RQ/Mili-Q system. Details regarding standard information and the selection of target compounds can be found in Table S1 and Text S1.

### 2.2. Study Area and Sample Collection

**Figure 1:**
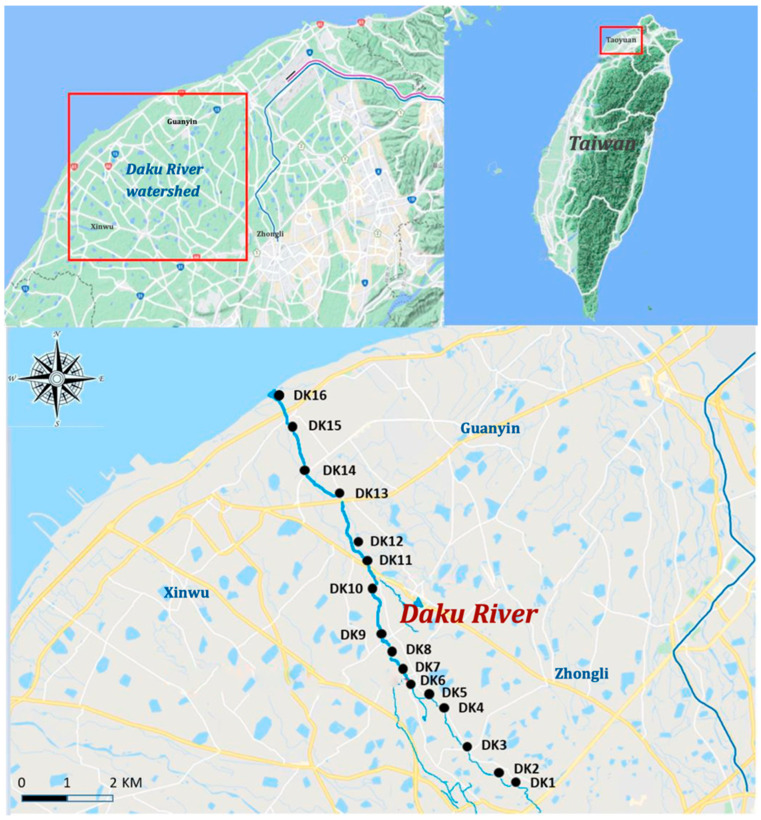
Study area and sampling locations.

Monthly water sampling was conducted from August 2019 to July 2020 along the Daku River, resulting in 12 sampling campaigns and a total of 192 water samples. A total of 16 sites were selected to represent the upper, middle, and lower sections of the river. Sampling locations were strategically placed before and after the confluence points with Litouzhou ditch and Wayao ditch to assess changes in PFAS concentrations across these junctions. Four downstream sites (DK13–DK16) adjacent to the Guanyin Industrial Zone were included to capture the potential industrial sources of PFASs (Figure 1). Surface water samples (1 L each) were collected in precleaned polypropylene bottles. At each site, three replicate samples were taken to account for spatial heterogeneity. The samples were maintained at 4 °C in the laboratory subsequent to transportation in insulated containers, pending further processing.

### 2.3. Sample Pretreatment

The sample preparation process followed established protocols, as outlined in previous studies [15,16]. Briefly, 1 L grab water samples were passed through 0.45 mm GF/F membranes. The resulting filtrate was then enhanced with isotopically labeled surrogates, ^13^C_8_-PFOA and ^13^C_9_-PFNA, to assess procedural recovery. A 500 mg 6 mL Oasis HLB solid-phase extraction (SPE) cartridge (Waters, Milford, MA, USA) underwent conditioning with 6 mL methanol and 6 mL deionized (DI) water. Water samples were subsequently introduced to the cartridge at approximately 15 mL/min. The cartridges were cleansed using 6 mL DI water and dried under vacuum in the SPE manifold. Elution of the samples was performed using 6 mL of methanol. The eluates were then evaporated to dryness under a gentle nitrogen stream and reconstituted to a final volume of 0.5 mL using a 50:50 (*v*/*v*) mixture of methanol and DI water. Prior to chemical analysis, the final solutions were filtered through a 0.22 mm nylon membrane filter and transferred to an amber autosampler vial.

### 2.4. Instrumental Analysis

An AB Sciex API 4000 QTRAP LC-MS/MS instrument (Applied Biosystems, Foster City, CA, USA) was utilized for sample analysis. Analyte separation was conducted using a ZORBAX Eclipse XDB-C18 column measuring 150 × 4.6 mm with a 5 mm particle size (Agilent, Palo Alto, CA, USA). The method employed a binary gradient with a 350 mL/min flow rate and a 50 mL injection volume. The mobile phases consisted of 0.1% formic acid for both A and B. Table S2 outlines the specific elution program conditions. An API 4000 triple quadrupole mass spectrometer (Applied Biosystems, USA) equipped with an electrospray ionization (ESI) interface operating in negative ion mode was used for mass spectrometric measurements. Target compounds were identified using two multiple reaction monitoring (MRM) pairs, with ions acquired in MRM mode. Tables S3 and S4 provide detailed information on the MS/MS parameters, conditions, and MRM pairs.

### 2.5. Quality Assurance and Quality Control (QA/QC)

To enhance the reliability of the experimental data, isotope standards of known concentrations were added to each sample, and the recovery rate was calculated to assess potential sample loss during the procedure (^13^C_8_-PFOA: 97 ± 12% and ^13^C_9_-PFNA: 101 ± 16%). To prevent contamination, a blank analysis was conducted after every two batches of experiments to ensure no interference or contamination occurred. For the blank analysis, 600 mL of Milli-Q deionized water was processed using the same protocol as for the samples. In this study, the method detection limit (MDL) was established using a signal-to-noise (S/N) ratio of 10. The MDL range was between 0.01 ng L^−1^ and 0.12 ng L^−1^, as outlined in Table S4.

### 2.6. Statistical Analysis

In this study, we employed principal component analysis (PCA) and positive matrix factorization (PMF) to explore and characterize the collected data. As an exploratory tool, PCA helps determine how many principal components (PCs) are required to account for the majority of the observed variance. We used varimax rotation in PCA, retaining only the largest coefficients in the varimax normalized matrix. Meanwhile, PMF is a multivariate factor analysis method that partitions the original data matrix into two outcomes: factor contributions and factor profiles. These outcomes can be examined alongside emission or discharge inventories to infer possible contributing sources. PMF implementation hinges on estimating the uncertainties associated with each data value; these uncertainties (u) effectively downweight missing data and values below the detection limit, thus refining the factorization process. Details regarding the matrix of measured uncertainties of PMF can be found in Text S2.

### 2.7. Risk Characterization

The evaluation of potential health risks to humans from PFAS exposure involved comparing measured levels with established guidelines and either the estimated daily intake (*EDI*) or the Reference Dose (*RfD*). To assess the risk associated with consuming contaminated crops, the researchers calculated the Health Risk Index (*HRI*) using Equation (1):

(1) $$HRI=\frac{EDI}{RfD}$$

This approach provides an assessment of the potential health risks for residents within the Daku River watershed. An HRI greater than 1 indicates a potential health risk, while an HRI less than or equal to 1 suggests negligible risk. The RfD is based on the reference dose of PFAS provided by EPA [17,18], and the RfD value for both PFOA and PFOS is 20 ng/kg bw/day [19]; the EDI was calculated as in Equation (2) below:

(2) $$EDI=\frac{C\times IR\times EF\times ED}{BW\times AT}$$

where C is the concentration of PFASs detected in crops; IR is the daily intake of greens or fruits and vegetables of Taiwanese residents (Table S5); EF is the exposure frequency over 365 days/year; ED is the exposure duration over 30 years [20]; BW is the average body weight in kg for residents of a specific age in Taiwan; and AT is the average exposure time over 365 days × ED [20].

Equation (3), as presented by [19], was utilized to determine the PFAS levels in the crop based on the PFAS concentrations found in the water.

(3) Cp=Cw×TF

where Cp is the concentration of PFASs in crops, Cw is the concentration of PFASs in water, and TF is the transfer factors of the targeted crops; these were found by searching the literature and are listed in Table S6.

## 3. Results and Discussion

### 3.1. Occurrence of PFASs in Daku River

In this study, water samples were collected monthly from August 2019 to July 2020 at 16 sites along the Daku River (Figure 1), covering the area from upstream to the downstream estuary. Ten per- and polyfluoroalkyl substances (PFASs) were analyzed, comprising three perfluoroalkyl sulfonates (PFSAs: PFBS, PFHxS, PFOS) and seven perfluoroalkyl carboxylates (PFCAs: PFHxA, PFHpA, PFOA, PFNA, PFDA, PFUnA, PFDoA). Among these, PFHxA and PFDoA were consistently below the method detection limit (MDL) over the 12-month period and were therefore excluded from further discussion. The remaining eight PFASs, detected in 192 water samples (Table S7), ranged from below MDL to 185 ng/L. Perfluorooctanoic acid (PFOA) exhibited the highest concentration, reaching up to 185 ng L^−1^, and, on average, accounted for 52% (mean 61.9 ng L^−1^) of the total PFAS load. Perfluorobutyl sulfonate (PFBS) and perfluorononanoic acid (PFNA) contributed 15% (17.6 ng/L) and 13% (15.8 ng/L), respectively, while perfluoroundecanoic acid (PFUnA) was the least abundant (1.64 ng/L on average). The concentrations, from lowest to highest, followed the sequence PFUnA < PFOS < PFHxS < PFDA < PFNA < PFBS < PFOA.

Table 1 presents a comparative analysis of PFAS contamination levels in the Daku River and other global water bodies. The results indicate that PFBS concentrations in the Daku River (n.d.–111.58 ng/L) are notably higher than the values reported in the Yangtze River, China (n.d.–41.9 ng/L), and the Huai River, China (n.d.–9.83 ng/L) [11,21]. Such elevated PFBS levels suggest substantial industrial or municipal inputs, highlighting the need for targeted source identification. The PFOS concentrations in the Daku River (n.d.–20.7 ng/L) were moderate relative to those measured in highly impacted rivers (e.g., up to 195.8 ng/L in the Maozhou River, China), yet align with the levels documented in the Alabama River, USA (10.65–19.55 ng/L) [22,23]. Although PFOS contamination is evident, it appears less severe than in more industrialized or densely populated basins. PFOA in the Daku River (0.08–185 ng/L) falls within a range comparable to that of the Alabama River (14.12–19.20 ng/L) and is somewhat lower than the maximum concentration (8.71–222.17 ng/L) reported near Shanghai Pudong International Airport, China [23,24]. This pattern implies that local industrial activities, potentially including fluoropolymer manufacturing and the use of aqueous film-forming foams (AFFFs), may be key contributors. The concentrations of PFNA (n.d.–67.76 ng/L) and PFDA (0.16–46.39 ng/L) in the Daku River exceed those in several Chinese rivers, such as the Maozhou and Huai (0.4–1.6 ng/L and 0.46–1.93 ng/L for PFNA), and the Changhua and Wanquan (0.00–0.08 ng/L and 0.02–0.13 ng/L for PFDA) [21,22,25]. These disparities suggest that industrial discharges or wastewater effluents may deliver significant PFNA and PFDA loads into the Daku River. Compared with reservoir environments, PFAS concentrations in the Baoshan Reservoir (e.g., PFOS at 0.02–61.2 ng/L) appear modest relative to those in heavily polluted rivers, indicating that lower industrial and municipal effluent inflows may help mitigate contamination in reservoir systems [16]. Overall, PFAS pollution in the Daku River exhibits concentrations on par with or exceeding those reported in other industrialized areas in Asia and the United States. Elevated levels of certain compounds, especially PFBS and PFOA, underscore the importance of continued monitoring and improved management strategies, particularly given their links to industrial processes, wastewater releases, and possible atmospheric inputs.

**Figure 2:**
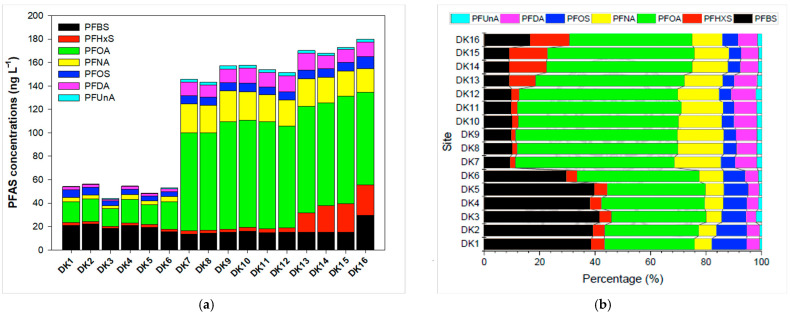
(**a**) Spatial distribution and (**b**) compositional profiles of PFASs in Daku River.

To investigate spatial variation, surface water samples were obtained from 16 sites along the 14.5 km main river channel (Figure 2). The total PFAS concentrations remained below 60.0 ng/L in the upper sections, but sharply increased to 146 ng/L at DK7, located near the confluence with Litouzhou ditch. This pattern suggests that higher PFAS loads in Litouzhou ditch significantly elevate concentrations downstream of the confluence. Specifically, four- to fivefold increases were observed for PFOA, PFNA, PFDA, and PFUnA after passing DK7, while PFBS remained relatively stable from upstream to downstream. Perfluorohexane sulfonate (PFHxS) showed a moderate increase toward the river mouth (DK13–DK16), implying additional emissions from nearby industrial operations. The concentrations of perfluorooctane sulfonate (PFOS) were relatively consistent throughout the river.

**Figure 3:**
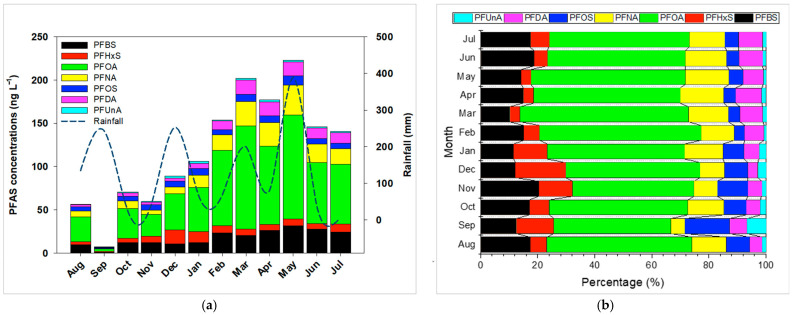
(**a**) Temporal distribution and (**b**) compositional profiles of PFASs in Daku River from August 2019 to July 2020.

Seasonal fluctuations in PFAS concentrations, illustrated in Figure 3, reveal higher levels between March and May (223 ng/L) and lower values (56.5 ng/L) from August to November. These findings align with reports from the Yangtze River, China [11], and may be attributed to complex interactions involving temperature, rainfall, and varying inputs of treated or untreated wastewater, as well as microbial activity in the water column. Furthermore, the reduced agricultural water supply from the Taoyuan Canal between March and May likely diminishes dilution capacity, thereby increasing PFAS concentrations. Monthly compositional data (Figure 3) highlight PFOA’s dominant contribution (41–59%), followed by PFBS (10–20%) and PFNA (5–15%). Perfluoroundecanoic acid (PFUnA) consistently contributed <6% each month. Overall, most PFAS exhibited relatively stable proportions over the year.

### 3.2. Source Identifications

**Figure 4:**
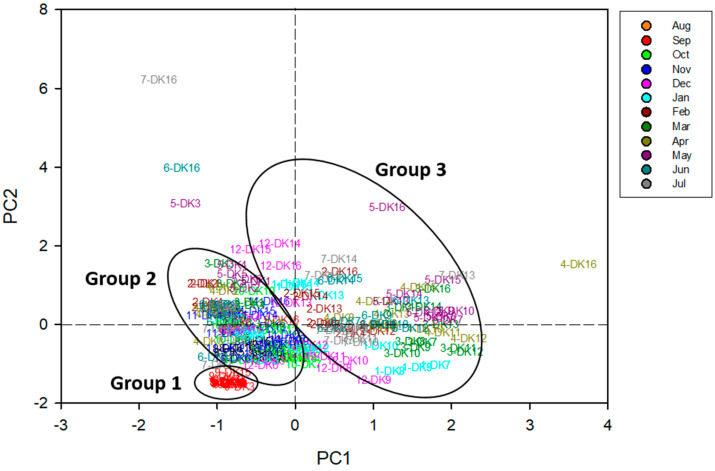
Principal component analysis (PCA) score plot produced in this study.

To elucidate the composition and possible origins of PFASs in the Daku River, principal component analysis (PCA) was performed on data from the 16 sampling sites, with non-detected values assigned as ½ of the detection limit (DL). The PCA outcome (Table S8) yielded two principal components (PC1 and PC2). The first two principal components (PCs), which collectively account for 76.1% of the variance in the original dataset, exhibited eigenvalues exceeding one. Specifically, the first principal component (PC1) accounted for 54.7% of the variance, while the second component accounted for 21.4%. Loading scores exceeding 0.32 were deemed significant. Examination of the component matrix (Table S9) indicated that PC1 was predominantly influenced by PFOA, PFNA, PFOS, PFDA, and PFUnA, whereas PFBS, PFHxS, and PFOS contributed most strongly to PC2. The spatial analysis further supports these factor loadings. Sites with elevated PC1 scores were situated downstream of the confluence between the Daku River and Litouzhou ditch, implying that PFOA, PFNA, PFOS, PFDA, and PFUnA likely entered the river from Litouzhou ditch. This pattern is consistent with industrial effluents, including fluorinated compound manufacturing [30], electronics production [31], and chemical or machinery operations [32]. In contrast, PC2, which was dominated by PFBS and PFHxS, with some PFOS, was more closely linked to agricultural activities [33] and potential wastewater discharge from the semiconductor industry [31]. The factor score plot (Figure 4) segregated samples into three groups: (i) Group 1, consisting of September samples with relatively lower PFAS levels, likely reflecting dilution by rainfall; (ii) Group 2, representing sites in the upper Daku River with moderate concentrations; and (iii) Group 3, covering locations near Litouzhou ditch, exhibiting higher PFAS burdens likely from industrial operations. It is worth noting that short-chain substances such as PFBS are increasingly used as substitutes for longer-chain PFASs, particularly where regulations limit the use of PFOS. The prominence of PFBS and PFHxS in PC2 suggests ongoing input from more recent industrial processes, including those associated with semiconductor manufacturing and agricultural supply chains that have switched to short-chain alternatives [33]. The sharp increase in PFAS load observed at DK7 can be largely attributed to industrial activities in the vicinity, particularly those associated with the Litouzhou ditch. As indicated by the PCA results (Group 3), locations near this ditch exhibit higher PFAS burdens, likely stemming from industrial operations. Mixed wastewater discharges from semiconductor plants, coating factories, and other industrial processes simultaneously release short-chain PFBS and PFHxS alongside longer-chain PFASs. The factor-based groupings and elevated PFAS concentrations near the Litouzhou ditch underscore the localized impact of these industrial sources. Moreover, such findings highlight the broader role of anthropogenic activities in shaping the overall PFAS burden.

To complement the PCA findings, positive matrix factorization (PMF) was conducted, producing three factors (Table S10) that further clarify potential sources: (i) Factor 1 is dominated by PFUnA, suggesting a link to anti-fouling or anti-grease agents found in food packaging, leather, and textile industries [34]. This aligns with processes where PFUnA-containing compounds are used for imparting water or grease resistance. (ii) Factor 2 is composed principally of PFBS, PFHxS, and PFOS (i.e., primarily perfluorosulfonic acids). These compounds may originate from local agricultural practices [35], where PFOS-related products were historically used for pest control or soil treatments, and from semiconductor-sector wastewater [31]. The prominence of PFBS implies the ongoing usage of short-chain alternatives in industrial settings. (iii) Factor 3 is characterized by PFOA, PFNA, and PFDA, implicating fluorinated compound manufacturing, electronics wastewater, and emissions from chemical, mechanical, or paint-producing facilities [32,36]. The presence of long-chain PFAS (e.g., PFOA) is particularly associated with processes such as fluoropolymer production and the formulation or use of aqueous film-forming foams (AFFFs).

Taken together, the PCA and PMF results converge on three principal PFAS source categories in the Daku River basin: (i) industrial effluents tied to electronics manufacturing, fluoropolymer production, and machinery/chemical operations; (ii) agricultural inputs, particularly in areas where PFOS or short-chain PFASs might be used or where legacy PFOS contamination persists; and (iii) mixed wastewater discharges from semiconductor plants, coating factories, or other industrial processes, which concurrently release short-chain PFBS and PFHxS alongside longer-chain PFASs. The factor-based groupings and the high PFAS levels near Litouzhou ditch emphasize the significance of localized industrial discharges. Meanwhile, the lower concentrations during rainfall events underline the importance of hydrological factors such as dilution and stormwater runoff in modulating PFAS contamination patterns. Continuous shifts toward short-chain substitutes are evident in the detection of PFBS and PFHxS, reinforcing the notion that monitoring programs should adapt to the evolving industrial usage of replacement PFASs.

From a management perspective, the identification of distinct source signatures underscores the need for targeted mitigation strategies. Industrial clusters requiring enhanced pretreatment of wastewater, agricultural areas potentially relying on PFAS-containing agents, and advanced semiconductor or electronics facilities all merit regulatory focus. Further research on the fate and transport of these compounds, including atmospheric deposition and infiltration into groundwater, will enable a more robust understanding of PFAS distribution in the Daku River watershed and facilitate the development of effective remediation and policy interventions.

### 3.3. Mass Loadings of PFASs

PFASs are known to persist in the aqueous phase due to their high chemical stability and solubility, which enables their downstream transport through riverine flows. Within the Daku River watershed, a key source region for PFASs, cumulative discharges into both riverine and marine environments were assessed using mass loading estimates. This was calculated using Equation (4) [37]:

Mass loading = C_water_ × F_water_ (4)

where C_water_ is the PFAS concentration in water (ng/L) and F_water_ represents the river discharge. For non-detected values, half of the method detection limit (MDL) was used as a surrogate concentration. Equation (4) is commonly used in PFAS load assessments because it directly combines the measured contaminant concentration with the volume of water flowing through the river, providing a straightforward method for quantifying the overall mass of PFASs transported downstream.

**Figure 5:**
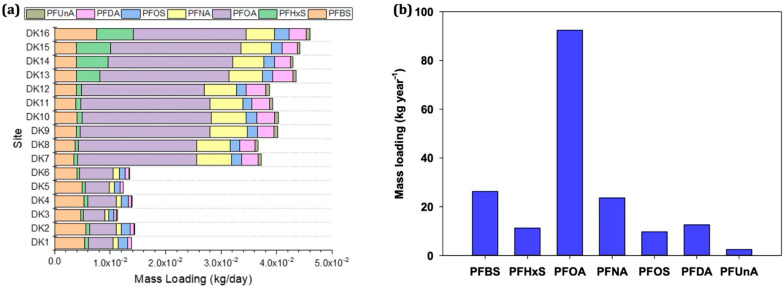
(**a**) Estimated mass loadings from August 2019 to July 2020 and (**b**) annual emissions of PFASs in Daku River.

Daily PFAS loadings in the Daku River ranged from 8.39 × 10^−5^ kg/day to 2.35 × 10^−2^ kg/day (Figure 5a). Among the detected compounds, PFOA accounted for the highest daily emissions (3.85 × 10^−3^ kg/day to 2.35 × 10^−2^ kg/day), followed by PFBS and PFNA, with loadings of 3.46 × 10^−3^ kg/day to 7.57 × 10^−3^ kg/day and 6.43 × 10^−4^ kg/day to 6.75 × 10^−4^ kg/day, respectively. Meanwhile, PFUnA exhibited the lowest daily load (8.39 × 10^−5^ kg/day to 6.84 × 10^−4^ kg/day). Spatially, PFAS fluxes increased substantially downstream of DK7, consistent with higher PFAS concentrations attributed to industrial discharges and municipal wastewater inputs. The greatest PFAS load occurred at DK16 near the river mouth (Figure 5a), underscoring the cumulative effect of upstream sources converging toward the estuary. Annual emissions (Figure 5b) further highlighted PFOA as the dominant contributor (92.4 kg year^−1^). PFBS followed at 26.3 kg year^−1^, then PFNA (23.6 kg year^−1^), PFDA (12.6 kg year^−1^), PFHxS (11.3 kg year^−1^), PFOS (9.70 kg year^−1^), and PFUnA (2.45 kg year^−1^). The prominence of PFOA in yearly flux aligns with global trends, wherein long-chain PFCAs remain high due to historical production volumes and ongoing usage in certain industrial processes. By contrast, PFBS, now widely employed as a substitute for PFOS, indicates a shift toward short-chain alternatives in multiple sectors, such as electronics, textile treatment, and metal plating.

These findings underscore the importance of anthropogenic activities, particularly fluoropolymer manufacturing, metal finishing operations, semiconductor industries, and possibly the use of AFFFs, in shaping the overall PFAS burden [32]. The substantial flux in downstream locations confirms that industrial clusters and wastewater outfalls near DK7 exert a strong influence on PFAS export to coastal zones. These results suggest that controlling PFAS emissions at upstream industrial sites could significantly reduce overall discharge to the Daku River and, ultimately, to coastal waters. Strategies such as advanced treatment processes (e.g., activated carbon, ion exchange, or high-pressure membranes) and improved industrial wastewater management may be particularly effective in mitigating PFAS release.

### 3.4. Health Risk Assessment

A prior study reported that approximately 16,778 CMD of industrial wastewater is discharged into the Daku River [38]. Given the unique physicochemical properties of PFASs used in industrial processes, these compounds often evade removal via conventional wastewater treatment and can thus enter irrigation channels. When absorbed by crops irrigated with this water, PFASs may accumulate in edible plant tissues, eventually posing human health risks through dietary intake.

To estimate PFAS concentrations in crops, we applied a transfer factor approach incorporating water-phase concentrations. The derived values for PFOA and PFOS are shown in Table S11. Among the investigated crop types, PFOA exhibited the highest accumulation in carrot, celery, wheat, and rice, whereas PFOS displayed relatively greater enrichment in carrot and celery. By contrast, tomatoes exhibited the lowest accumulation factors for both compounds. Health risks associated with PFOA and PFOS in the Daku River watershed were assessed by combining crop-specific PFAS concentrations, vegetable intake levels, and average body weights across different sexes and age groups (Table S12). Overall, tomatoes contributed the lowest PFAS concentrations, while the PFOA levels surpassed PFOS in all crops except celery. Notably, the estimated daily intake (EDI) values for males aged 13–18 were minimal for all crops except rice—likely reflecting lower per capita consumption of other vegetables—while females aged 13–18 showed the lowest EDI values except for wheat. Additionally, rice emerged as a prominent source of PFOA and PFOS for males, whereas tomato intake yielded relatively higher PFAS exposure in females.

The hazard indices (HIs) for PFOA and PFOS were determined based on reference doses (RfDs) of 20 ng kg^−1^ day^−1^, as recommended by the U.S. EPA [17,18]. The calculated HIs (Table S13) indicated that tomatoes carried the lowest health risk for both compounds, whereas rice posed the highest PFOA risk, and celery accounted for the highest PFOS risk. Nevertheless, for all examined demographics, the hazard ratios (HRs) remained below 1, implying minimal acute health concerns from crop consumption in the Daku River region.

Despite these findings, PFASs exhibit persistence, resistance to environmental degradation, and high bioaccumulative potential over prolonged exposures. Their extensive environmental half-lives (often exceeding 40 years) and slow elimination in the human body can elevate long-term health risks. For example, PFOS and PFOA display respective biological half-lives of 5.4 and 3.4 years [39]. Consequently, sustained dietary intake of contaminated crops may progressively increase body burdens. Moreover, PFBS, the second most abundant PFAS detected in Daku River water, was not analyzed in crops; this gap in knowledge limits our understanding of its potential contribution to cumulative exposure. Although PFBS is a short-chain PFAS that can be metabolized, its possible health implications remain underexplored. Future research should therefore examine the levels of PFBS in agricultural produce and evaluate its fate in crop systems, thereby providing a more comprehensive assessment of exposure risks and informing appropriate mitigation strategies.

## 4. Conclusions

This study provides a comprehensive overview of PFASs in the Daku River watershed, highlighting their spatial and temporal distributions, emission loadings, and associated human health risks. Monthly sampling from 16 sites between August 2019 and July 2020 revealed that PFOA was the predominant PFAS, accounting for over half of the total PFAS burden. Marked concentration increases were observed downstream of the confluence with Litouzhou ditch, underscoring the significant influence of industrial inputs, especially from the fluorinated compound manufacturing and electronics sectors. Seasonal variations further indicated elevated PFAS levels during March to May, potentially linked to reduced dilution capacity due to reduced agricultural water supply. PCA and PMF both confirmed the coexistence of industrial, agricultural, and semiconductor-related PFAS sources. Mass loading calculations estimated that PFOA contributed the highest daily and annual emissions, followed by PFBS and PFNA. These loadings emphasize the risk of ongoing downstream and coastal contamination, potentially impacting sensitive marine environments. Health risk assessments, incorporating crop-specific PFAS concentrations and dietary habits, showed that the HIs for PFOA and PFOS remained below unity for all demographic groups, suggesting minimal immediate risk. However, the persistence and bioaccumulation potential of PFASs, as evidenced by their long environmental and biological half-lives, raise concerns regarding chronic exposures. Continuous monitoring, coupled with more advanced wastewater treatment processes, is therefore recommended to mitigate PFAS inputs into the watershed and protect human and ecological health. In particular, further studies should address emerging short-chain PFASs (e.g., PFBS) in agricultural products, given their possible contribution to cumulative exposure over time.

## Figures and Tables

**Table 1:** Global comparison of PFAS concentrations (ng/L).

Sampling Location	Location Type	Concentration (ng/L)							References
		PFBS	PFHxS	PFOS	PFHpA	PFOA	PFNA	PFDA	
Daku River, Taiwan	River	n.d.–111.58	n.d.–56.5	n.d.–20.7	n.d.–47.1	0.08–185	n.d.–67.76	0.16–46.39	This study
Yangtze River, China	River	n.d.–41.9	n.d.–18.0	n.d.–3.93	n.d.–2.59	0.52–18.0	n.d.–0.86	n.d.–0.33	[11]
Red River, Vietnam	River		n.d.	n.d.–0,22	n.d.–0.94	n.d.–1.51	n.d.–0.51	n.d.–0.61	[26]
Ganges River, India	River	n.d.–10.2	n.d.–0.304	n.d.–1.73	0.340–3.27	0.079–1.18	n.d.–0.19	n.d.–0.19	[27]
Maozhou River, China	River	16.4–42.5	0.13–11.9	1.7–195.8	1.5–17.0	3.2–27.0	0.4–1.6	0.18–0.83	[22]
Baoshan reservoir, Taiwan	Reservoir	0.02–7.94	n.d.–30.5	0.02–61.2	n.d.	0.02–68.9	0.02–33.4	n.d.	[16]
Alabama River, US (Mean ± SD)	River	28.24 ± 2.31–45.06 ± 3.00		10.65 ± 1.01–19.55 ± 2.14	7.55 ± 0.38–9.43 ± 0.78	14.12 ± 0.39–19.20 ± 1.52			[23]
Tunjuelo River, Colombia	River	0.08	0.52	0.24	0.03	0.06	<0.02	<0.02	[28]
River around Shanghai Pudong Intl’ Airport, China	River	n.d.–37.08	2.31–12.91	0.83–6.72	1.32–14.68	8.71–222.17	0.59–3.86	0.62–2.33	[24]
Huai River, China (Mean ± SD)	River	n.d.–9.83 (1.80 ± 1.57)	n.d.–4.76 (1.08 ± 0.89)	0.14–3.42 (1.21 ± 0.66)	0.06–5.94 (3.21 ± 1.21)	4.62–81.31 (16.85 ± 12.90)	0.46–1.93 (1.10 ± 0.30)	n.d.–0.61 (0.22 ± 0.12)	[21]
Southern Lyon, France	River	0.064–9.9	0.053–19	0.052–76	0.07–62	0.071–61	0.05–14	0.034–1.8	[29]
Nandu River, China	River	0.01–0.89	0.01–0.89	0.00–0.39	0.00–1.61	0.35–0.72	0.11–0.41	0.02–0.10	[25]
Changhua River, China	River	0.01–0.41	0.00–0.34	0.02–0.57	0.02–3.10	0.30–3.36	0.11–0.33	0.00–0.08	[25]
Wanquan River, China	River	0.04–1.17	0.00–0.85	0.04–1.48	0.12–1.21	0.35–8.55	0.14–0.35	0.02–0.13	[25]

n.d. = not detected; blank = not analyzed.

## Data Availability

The data are contained within the article.

## Supplementary Materials

The following supporting information can be downloaded at: https://www.mdpi.com/article/10.3390/toxics13060435/s1, Text S1: Selection of targeted PFAS compounds; Text S2: Matrix of measured uncertainties of PMF; Table S1: Information on 10 target analytes in this study; Table S2: Gradient elution program of chromatographic separation; Table S3: The LC-MS/MS operating parameters; Table S4: Method detection limits and MRM pairs for PFASs; Table S5: Daily vegetable intake in Taiwan by sex and age; Table S6: TF values of target crops; Table S7: Summary of results for detected PFASs in Daku River; Table S8: Principal component analysis of Daku River; Table S9: I PC loadings table after principal component analysis of Daku River; Table S10: PMF factor composition of PFASs (%); Table S11: Concentration of PFASs in target crops; Table S12: estimated daily intake of PFASs from crops in Daku River; Table S13: Hazard indices of PFASs from crops in Daku River. References [3,40,41,42,43,44,45,46] are cited in the supplementary materials.
