# Supplementary material for: Comprehensive Risk Evaluation of Perfluoroalkyl Substance Pollution in Urban Riverine Systems: Ecotoxicological and Human Health Perspectives
Source: Toxics. 2025 May 26;13(6):435. doi: 10.3390/toxics13060435 (PMC12196852; doi:10.3390/toxics13060435)
Supplement: Supplementary file 1 [file toxics-13-00435-s001.zip › toxics-3474805-supplementary.pdf]

Supplementary Information

Comprehensive Risk Evaluation of PFAS Pollution in Urban Riverine Systems: Ecotoxicological and Human Health Perspectives

Contents

Text S1. Selection of the targeted PFAS compounds.

Text S2. The matrix of measured uncertainties of PMF.

Table S1. Information on the 10 target analytes in this study.

Table S2. Gradient elution program of chromatographic separation.

Table S3. LC-MS/MS operating parameters.

Table S4. Method detection limits, recovery rates and MRM pairs for PFASs.

Table S5. Daily Vegetable Intake in Taiwan by Sex and Age.

Table S6. TF values of target crops.

Table S7. Summary of results for the detected PFASs in Daku River.

Table S8. Principal Component Analysis of Daku River.

Table S9. PC loadings table after the principal component analysis of Daku River.

Table S10. PMF factor composition of PFASs (%).

Table S11. Concentration of PFASs in target crops.

Table S12. Estimated Daily Intake of PFASs from Crops in Daku River.

Table S13. Hazard indices of PFASs from Crops in Daku River.

Text S1. Selection of the targeted PFAS compounds.
It would be best to use as many PFAS standards as possible to provide a much broader data on PFAS investigation. However, due to the scale of the analytical lab, using a broader target compound were beyond our means. Another consideration is the fact that except for PFOA, PFNA and PFOS, other PFASs are not intentionally produced in large amount [1,2], most of which are emitted by impurities. Those PFASs are most likely under the detection limits, especially for samples in this study. Therefore, based on the detection frequency of PFASs in most published data in literatures, ten presentative PFASs and two isotope standards were selected in this study.

Text S2. The matrix of measured uncertainties of PMF.
The matrix of measured uncertainties is calculated using the error and the method detection limit (MDL) for each compound. Each species of calculated uncertainty was determined by the LOD, thus the LOD value of PFAS compounds was used as MDL value in this study. In cases where concentration ≤ MDL, the uncertainty (u) calculated as:

$$u = \frac{5}{6} MDL \quad (1)$$

When concentration > MDL, u is calculated as:

$$u = \sqrt{(ErrorFraction \times Concentration)^2 + (0.5 \times MDL)^2} \quad (2)$$

Based approximately on the Relative Standard Deviations (RSDs), the errors of all species were set at 0.05, and the additional modeling uncertainty was set at 5%. The robust Q value was calculated by excluding outliners, defined as samples for which the uncertainty-scaled residual exceeded 3. The true Q value was calculated including all points. Different runs were undertaken to boost the results by weighting the low S/N species as “strong” or "weak." When weighted as "strong", each of the six variables was included; when weighted “weak”, the sum of the seven PFASs (Σ_7PFASs) was used instead. The factor number (P) was gradually adapted from 2 to 4. Increasing P only assisted if there was a significant decrease in Q. The number of sources was determined by evaluating each source together with its best match, and the most reasonable results were considered.

Table S1. Information on the 10 target analytes in this study.

Compound	Number of Carbons	MW (g/mol)	log K_{ow}	CAS NO	Chemical Formula
Perfluoroalkyl sulfonic acids (PFSAs)					
Perfluorobutyl sulfonate (PFBS)	C4	300.1	2.63[a]	375-73-5	$C_4F_9SO_3^-$
Perfluorohexyl sulfonate (PFHxS)	C6	400.12	3.16[b]	355-46-4	$C_6F_{13}SO_3^-$
Perfluorooctyl sulfonate (PFOS)	C8	500.13	5.43[a]	1763-23-1	$C_8F_{17}SO_3^-$
Perfluoroalkyl carboxylic acids (PFCAs)					
Perfluorohexanoic acid (PFHxA)	C6	314.05	3.48[b]	307-24-4	$C_6F_{11}O_2^-$
Perfluoroheptanoic acid (PFHpA)	C7	364.06	4.41[a]	375-85-9	$C_7F_{13}O_2^-$
Perfluorooctanoic acid (PFOA)	C8	414.07	5.11[a]	335-67-1	$C_8F_{15}O_2^-$
Perfluorononanoic acid (PFNA)	C9	464.08	5.48[b]	375-95-1	$C_9F_{17}O_2^-$
Perfluorodecanoic acid (PFDA)	C10	514.08	6.51[a]	335-76-2	$C_{10}F_{19}O_2^-$
Perfluoroundecanoic acid (PFUnA)	C11	564.09	7.15[b]	2058-94-8	$C_{11}F_{21}O_2^-$
Perfluorododecanoic acid (PFDoA)	C12	614.1	7.49[b]	307-55-1	$C_{12}F_{23}O_2^-$

[a] [3]
[b] [4]

Table S2. Gradient elution program of chromatographic separation.

Effluent	Mobile phase A	0.1% formic acid in deionized water
	Mobile phase B	0.1% formic acid in methanol
	Flow rate	350 μL min^{-1}
Time (min)	Mobile phase	
	A (%)	B (%)
0.0	70	30
0.1	70	30
1.5	20	80
4.0	5	95
9.0	5	95
9.2	70	30
13	70	30

Table S3. LC-MS/MS operating parameters.

	ESI Negative Mode
Dwell time	50 ms
Ion Spray Voltage (S)	-4.5 kV
Curtain Gas (CUR)	10 L h-1
Gas 1 (GS1)	50 L h-1
Gas 2 (GS2)	60 L h-1
Temperature	500 ℃
Interface Heater (ihe)	ON
Collisionally Activated Dissociation	5

Table S4. Method detection limits, recovery rates and MRM pairs for PFASs.

Chemical	MDL (ng L^{-1})	Recovery rates (%)	MRM1 (quantification)	MRM2 (confirmation)
PFBS	0.02	92±13	299/80	299/99
PFHxS	0.03	89±19	399/80	399/99
PFOS	0.01	102±15	499/80	499/99
PFHpA	0.04	113±9	363/319	363/169
PFHxA	0.12	90±4	313/269	313/119
PFOA	0.01	107±17	413/369	413/169
PFNA	0.02	110±8	463/419	463/219
PFDA	0.01	99±10	513/469	513/269
PFUnA	0.03	84±11	563/519	563/269
PFDoA	0.05	96±14	613/569	613/269

Table S5. Daily Vegetable Intake in Taiwan by Sex and Age.

Gender	Age (years)	Average weight [a] (kg)	Average daily intake/day [b]				
			Tomato	Carrot	Celery	Wheat	Rice
Male	13-18	63.7	83.47	36.78	20.35	41.08	140.9
	19-64	70.4	207.9	62.45	33.46	94.55	149.9
	65-	65.0	86.87	78.36	36.36	51.07	152.6
Female	13-18	53.6	101.6	28.45	11.93	56.16	72.79
	19-64	57.0	188.7	54.60	32.23	42.46	87.97
	65-	56.6	131.1	48.77	30.26	43.89	114.2

[a] [5]
[b] [6]

Table S6. TF values of target crops.

Crop	Unit of measure	PFOA	PFOS
Tomato	L/kg	0.33[a]	0.03[a]
Carrot	L/kg	1.48[a]	9.91[a]
Celery	L/kg	2.46[a]	59.7[a]
Wheat	L/kg	2.09[b]	1.00[b]
Rice	L/kg	1.60[b]	0.20[b]

[a] [7]
[b] [8]

Table S7. Summary of results for the detected PFASs in Daku River.

	N	Concentration (ng L^{-1})		
		Min	Max	Mean
PFBS	192	0.04	112	17.6
PFHxA	192	0	0	0
PFHpA	192	0	47.1	15.0
PFHxS	192	0	56.5	7.53
PFOA	192	0.08	185	61.9
PFNA	192	0	67.8	15.8
PFOS	192	0	20.7	6.49
PFDA	192	0.16	46.4	8.42
PFUnA	192	0	7.61	1.64
PFDoA	192	0	0	0

Table S8. Principal Component Analysis of Daku River.

Total variation									
Component	Initial intrinsic value			Extract the square and the amount of load			Rotation squared and loaded		
	Total	% of variation	Cumulative %	Total	% of variation	Cumulative %	Total	% of variation	Cumulative %
1	4.287	61.25	61.25	4.287	61.25	61.25	3.830	54.72	54.72
2	1.040	14.86	76.11	1.040	14.86	76.11	1.497	21.39	76.11
3	0.924	13.20	89.30						
4	0.399	5.695	95.00						
5	0.257	3.666	98.67						

Table S9. PC loadings table after the principal component analysis of Daku River.

Principal Component Matrix		
Compound	Component	
	1	2
PFBS	0.018	0.852
PFHxS	0.214	0.575
PFOA	0.917	0.251
PFNA	0.942	0.207
PFOS	0.656	0.513
PFDA	-.917	0.268
PFUnA	0.886	-0.019

Table S10. PMF factor composition of PFASs (%).

PFASs	Factor 1	Factor 2	Factor 3
PFBS	1.56	49.46	48.98
PFHxS	0.78	65.95	33.27
PFOA	0.13	26.47	73.40
PFNA	0.01	22.49	77.50
PFOS	4.02	95.97	0.00
PFDA	4.90	19.82	75.28
PFUnA	82.56	15.29	2.15

Table S11. Concentration of PFASs in target crops.

Crop	Unit of measure	PFOA	PFOS
Tomato	ng/kg	20.4	0.20
Carrot	ng/kg	91.6	64.3
Celery	ng/kg	152	387
Wheat	ng/kg	129	6.49
Rice	ng/kg	99.0	1.30

Table S12. Estimated Daily Intake of PFASs from Crops in Daku River.

Gender	Age (years)	EDI									
		Tomato		Carrot		Celery		Wheat		Rice	
		PFOA	PFOS	PFOA	PFOS	PFOA	PFOS	PFOA	PFOS	PFOA	PFOS
Male	13-18	2.67 x 10^{-2}	2.62 x 10^{-4}	5.29 x 10^{-2}	3.71 x 10^{-2}	4.86 x 10^{-2}	0.12	8.34 x 10^{-2}	4.19 x 10^{-3}	0.22	2.88 x 10^{-3}
	19-64	6.02 x 10^{-2}	5.91 x 10^{-4}	8.13 x 10^{-2}	5.70 x 10^{-2}	7.23 x 10^{-2}	0.18	0.17	8.73 x 10^{-3}	0.21	2.77 x 10^{-3}
	65-	2.73 x 10^{-2}	2.67 x 10^{-4}	0.11	7.75 x 10^{-2}	8.51 x 10^{-2}	0.22	0.10	5.11 x 10^{-3}	0.23	3.05 x 10^{-3}
Female	13-18	3.87 x 10^{-2}	3.79 x 10^{-4}	4.86 x 10^{-2}	3.41 x 10^{-2}	3.39 x 10^{-2}	0.09	0.14	6.81 x 10^{-3}	0.13	1.77 x 10^{-3}
	19-64	6.75 x 10^{-2}	6.62 x 10^{-4}	8.77 x 10^{-2}	6.16 x 10^{-2}	8.61 x 10^{-2}	0.22	9.63 x 10^{-2}	4.84 x 10^{-3}	0.15	2.01 x 10^{-3}
	65-	4.73 x 10^{-2}	4.63 x 10^{-4}	7.89 x 10^{-2}	5.54 x 10^{-2}	8.14 x 10^{-2}	0.12	0.10	5.04 x 10^{-3}	0.20	2.62 x 10^{-3}

Table S13. Hazard indices of PFASs from Crops in Daku River.

Gender	Age (years)	HIs									
		Tomato		Carrot		Celery		Wheat		Rice	
		PFOA	PFOS	PFOA	PFOS	PFOA	PFOS	PFOA	PFOS	PFOA	PFOS
Male	13-18	1.34 x 10^{-3}	1.31 x 10^{-5}	2.64 x 10^{-3}	1.86 x 10^{-3}	2.43 x 10^{-3}	6.18 x 10^{-3}	4.17 x 10^{-3}	2.10 x 10^{-4}	1.09 x 10^{-2}	1.44 x 10^{-4}
	19-64	3.01 x 10^{-3}	2.95 x 10^{-5}	4.06 x 10^{-3}	2.85 x 10^{-3}	3.62 x 10^{-3}	9.20 x 10^{-3}	8.68 x 10^{-3}	4.36 x 10^{-4}	1.05 x 10^{-2}	1.38 x 10^{-4}
	65-	1.36 x 10^{-3}	1.34 x 10^{-5}	5.52 x 10^{-3}	3.88 x 10^{-3}	4.26 x 10^{-3}	1.08 x 10^{-3}	5.08 x 10^{-3}	2.55 x 10^{-4}	1.16 x 10^{-2}	1.53 x 10^{-4}
Female	13-18	1.93 x 10^{-3}	1.90 x 10^{-5}	2.43 x 10^{-3}	1.71 x 10^{-3}	1.69 x 10^{-3}	4.31 x 10^{-3}	6.77 x 10^{-3}	3.41 x 10^{-4}	6.72 x 10^{-2}	8.83 x 10^{-4}
	19-64	3.38 x 10^{-3}	3.31 x 10^{-5}	4.39 x 10^{-3}	3.08 x 10^{-3}	4.30 x 10^{-3}	1.09 x 10^{-3}	4.82 x 10^{-3}	2.42 x 10^{-4}	7.64 x 10^{-2}	1.00 x 10^{-4}
	65-	2.36 x 10^{-3}	2.32 x 10^{-5}	3.95 x 10^{-3}	2.77 x 10^{-3}	4.07 x 10^{-3}	1.03 x 10^{-3}	5.01 x 10^{-3}	2.52 x 10^{-4}	9.99 x 10^{-2}	1.31 x 10^{-4}

References

1. Prevedouros, K.; Cousins, I.T.; Buck, R.C.; Korzeniowski, S.H. Sources, Fate and Transport of Perfluorocarboxylates. *Environ. Sci. Technol.* **2006**, *40*, 32–44, doi:10.1021/es0512475.

2. Wang, Z.; Cousins, I.T.; Scheringer, M.; Buck, R.C.; Hungerbühler, K. Global Emission Inventories for C4-C14 Perfluoroalkyl Carboxylic Acid (PFCA) Homologues from 1951 to 2030, Part I: Production and Emissions from Quantifiable Sources. *Environ. Int.* **2014**, *70*, 62–75, doi:10.1016/j.envint.2014.04.013.

3. Park, M.; Daniels, K.D.; Wu, S.; Ziska, A.D.; Snyder, S.A. Magnetic Ion-Exchange (MIEX) Resin for Perfluorinated Alkylsubstance (PFAS) Removal in Groundwater: Roles of Atomic Charges for Adsorption. *Water Res.* **2020**, *181*, 115897, doi:10.1016/j.watres.2020.115897.

4. National Library of Medicine PubChem Available online: https://pubchem.ncbi.nlm.nih.gov/.

5. Surveillance and Epidemiology Branch Center for Health Protection Departement of Health *Report of the 2014-2015 Demographic Health Survey*; 2017;

6. Food and Drug Administration; National Health Research Institute National Food Consumption Database Available online: https://tnfcds.nhri.edu.tw/.

7. Arcadis U.S. Inc. *Review of Models for Evaluating Per- and Polyfluoroalkyl Substances in Land Applied Residuals and Biosolids*; v. 1.1.; National Council for Air and Stream Improvement, Inc.: Cary, U.S., 2020;

8. Costello, M.C.S.; Lee, L.S. Sources, Fate, and Plant Uptake in Agricultural Systems of Per- and Polyfluoroalkyl Substances. *Curr. Pollut. Reports* **2024**, *10*, 799–819, doi:10.1007/s40726-020-00168-y.
